# Hepatocellular carcinoma in patients with nonalcoholic fatty liver disease: A systematic review and meta-analysis: HCC and Steatosis or Steatohepatitis

**DOI:** 10.1016/j.neo.2022.100809

**Published:** 2022-05-27

**Authors:** Fausto Petrelli, Michele Manara, Silvia Colombo, Gabriella De Santi, Michele Ghidini, Marco Mariani, Alessandro Iaculli, Emanuele Rausa, Valentina Rampulla, Marcella Arru, Matteo Viti, Veronica Lonati, Antonio Ghidini, Andrea Luciani, Antonio Facciorusso

**Affiliations:** a Oncology Unit, ASST Bergamo Ovest, Treviglio BG, Italy; b Surgery Unit, ASST Bergamo Ovest, Treviglio BG, Italy; c Hepatology Unit, ASST Bergamo Ovest, Treviglio BG, Italy; d Medical Oncology Unit, Fondazione IRCCS Ca' Granda Ospedale Maggiore Policlinico, Milan, Italy; e Oncology Unit, ASST Bergamo Ovest, Seriate BG, Italy; f General Surgery 1, Papa Giovanni XXIII Hospital, Bergamo, Italy; g Oncology Unit, Casa di cura Igea, Milano, Italy; h Gastroenterology Unit, Department of Surgical and Medical Sciences, University of Foggia, 71122 Foggia, Italy

**Keywords:** Nonalcoholic fatty liver disease, Steatosis, NASH, Hepatocellular carcinoma, Meta-analysis

## Abstract

**Background and aims:**

Hepatic steatosis of nonalcoholic etiology (nonalcoholic fatty liver disease; NAFLD) is an emergent condition that may lead to hepatic cirrhosis and finally to liver cancer. We evaluate the risk of developing hepatocellular carcinoma (HCC) and quantify the prognosis in terms of recurrence (DFS) as well as HCC-specific and overall survival (CSS and OS) of patients with and without NAFLD.

**Methods:**

We searched published articles that evaluated the risk and outcomes of HCC in patients with steatosis/steatohepatitis from inception to July 2021 were identified by searching the PubMed, EMBASE, and Cochrane Library databases. Prospective cohort, case-control, or retrospective studies were selected that were published in English and provided incidence and survival rates of HCC patients with NAFLD. A random-effects model was created to estimate the pooled effect size. The primary outcome of interest was HCC incidence. The secondary endpoints were DFS, CSS, and OS.

**Results:**

In total, 948 217 patients with NAFLD were analyzed, from n = 103 observational studies. NAFLD significantly increased the risk of HCC (HR = 1.88 [95% CI, 1.46-2.42]; *P* < .01] but not risk of recurrence (HR = 0.99 [95% CI, 0.85-1.15]; *P* = .9) or overall mortality (HR = 1.04 [95% CI, 0.88-1.24]; *P* = 0.64). Conversely, NAFLD increased HCC-related mortality risk (HR = 2.16 [95% CI, 0.85-5.5]; P = .1). Risk of HCC was increased in Western countries but not in Asian countries.

**Conclusions:**

Patients with NAFLD have an increased risk of HCC as compared to patients without NAFLD. NAFLD also increases liver cancer (HCC) mortality. These results justify applying general measures to patients with proven NAFLD and monitoring patients with NASH and fibrosis.

## Highlights

• This systematic review and meta-analysis of 103 studies comprising 948 217 patients found that NAFLD is associated with a significantly higher risk of HCC as compared to no NAFLD.
• In addition, NAFLD nonsignificantly increased the risk of HCC-related mortality but not of recurrence or overall mortality.
• Given the higher risk of HCC in patients with NAFLD, general health interventions and screening should be implemented for high-risk cases (eg, those with steatohepatitis and fibrosis).

## Introduction

Hepatic steatosis of nonalcoholic etiology (nonalcoholic fatty liver disease or NAFLD) is an emergent condition that may lead to hepatic cirrhosis and finally to liver cancer. To define NAFLD, there must be evidence of hepatic steatosis and an absence of secondary causes of fat accumulation in the liver (eg, alcohol consumption). In most cases, NAFLD is commonly associated with metabolic comorbidities such as obesity, diabetes mellitus, and dyslipidemia. NAFLD comprises simple steatosis or steatohepatitis (NASH), where steatosis is associated with liver inflammation, with or without liver fibrosis [1]. The global incidence of NAFLD is rising in both Western and Asian countries due to the metabolic increase of etiological factors (eg, diabetes and obesity) [2], [3].

There is no specific therapy for NAFLD or screening method for at-risk patients; general health suggestions (eg, weight loss) are the only possible way to avoid or reduce the risk of NAFLD progression in fibrosis patients. An estimated 20% of patients with NASH will develop cirrhosis, and NASH is predicted to become the leading indication for liver transplants in the United States.

Nonalcoholic fatty liver disease with associated cirrhosis is a risk factor for the development of HCC. In a previous systematic review of 61 studies of patients with steatosis or NASH, the risk of HCC among those without and with cirrhosis ranged from 0.03 to 3.78 × 100 000 person-years [4]. Among subjects without cirrhosis, the risk of mortality from HCC was 0%-3% after longer observation. Furthermore, NASH is associated with liver-associated and overall mortality [5].

We performed an updated meta-analysis to verify the correlation and the prognostic significance of NAFLD in patients with HCC.

## Materials and Methods

To comprehensively calculate the cumulative incidence and prognosis of HCC in patients with NAFLD, a systematic review was conducted following the Preferred Reporting Items for Systematic Reviews and Meta-Analyses (PRISMA) guidelines.

### Eligibility Criteria for the Studies

All articles reporting the risk of HCC as a complication of NAFLD were included. NAFLD cases were defined by a positive biopsy for steatosis or by a suspect radiology examination of the liver. All cross-sectional, retrospective, and prospective studies that included patients with NAFLD and reported incidence of HCC were considered eligible. Case reports with fewer than 10 patients and case series, including all editorials, reviews, and commentaries, were excluded. Studies targeting special populations such as pregnant women, children, and other groups, were excluded. Only articles written in English were included.

### Search Strategy

Three bibliographical databases (PubMed, EMBASE, and the Cochrane Library) were searched to identify potential articles (as of July 31, 2021). The search criteria were as follows: *((("liver fatty"[All Fields] OR ("naflds"[All Fields] OR "non alcoholic fatty liver disease"[MeSH Terms] OR ("non alcoholic"[All Fields] AND "fatty"[All Fields] AND "liver"[All Fields] AND "disease"[All Fields]) OR "non alcoholic fatty liver disease"[All Fields] OR "nafld"[All Fields]) OR ("fatty liver"[MeSH Terms] OR ("fatty"[All Fields] AND "liver"[All Fields]) OR "fatty liver"[All Fields] OR "steatohepatitis"[All Fields]) OR ("fatty liver"[MeSH Terms] OR ("fatty"[All Fields] AND "liver"[All Fields]) OR "fatty liver"[All Fields] OR "steatosis"[All Fields]) OR "nash"[All Fields]) AND ("hcc"[All Fields] OR "HEPATOCELLULAR"[All Fields]) AND ("cancer s"[All Fields] OR "cancerated"[All Fields] OR "canceration"[All Fields] OR "cancerization"[All Fields] OR "cancerized"[All Fields] OR "cancerous"[All Fields] OR "neoplasms"[MeSH Terms] OR "neoplasms"[All Fields] OR "cancer"[All Fields] OR "cancers"[All Fields] OR ("carcinoma"[MeSH Terms] OR "carcinoma"[All Fields] OR "carcinomas"[All Fields] OR "carcinoma s"[All Fields])) AND "english"[Language])*

### Data Extraction and Inclusion Criteria

Data were extracted from the articles and supplementary materials. Reference lists from the eligible articles were retrieved to obtain further relevant studies. Duplicates between the databases were removed. To identify eligible studies, the retrieved articles were screened based on their title and abstract. Then, the potentially eligible studies were fully reviewed by 2 authors (AG and FP). Information was collected on the study characteristics, country, study design, follow-up, number of patients with NAFLD, number of patients with HCC, and NAFLD characteristics (such as type, diagnosis other than HCC stage, and outcome).

### Endpoints and Statistical Analysis

The primary endpoints were (a) the global incidence of HCC in NAFLD patients and (b) the association of NAFLD with the risk of HCC. The secondary endpoints were the associations of HCC with relapse (DFS), cancer mortality (CSM), and all-cause mortality (OS).

Critical assessment was conducted of the study setting and SARS-CoV-2 diagnosis to reduce the bias. The Newcastle–Ottawa scale (NOS) was used as a critical appraisal tool with which to assess the quality of the eligible studies.

The cumulative incidence rate of HCC was calculated for NAFLD cases by dividing the number of NAFLD cases with HCC by the total number of NAFLD cases, which was expressed as a percentage (%) with 95% confidence intervals (95% CI). Pooled odds ratios (HRs) and 95% CIs were calculated to assess the association of NAFLD with the occurrence of HCC, as compared to non-NAFLD subjects. Similarly, HRs for DFS, CSM, and OS were calculated to correlate HCC with the outcome. The pooled HRs and 95% CIs are presented in a forest plot.

Metaregression analyses were also performed for the primary analysis according to steatosis/NASH, cirrhosis, and hepatitis B/C rate among patients as well as race, duration of follow-up, and type of study.

*Z* tests were performed to assess the association between HCC and the presence of NAFLD (*P* < .05 was considered statistically significant). *Q* tests were used to evaluate the heterogeneity among studies, and the data with heterogeneity were analyzed using a random effects model. The publication bias was assessed using Egger's test and a funnel plot (*P* < 0.05 for Begg's test was considered having potential for publication bias). The data were analyzed using Review Manager, version 5.3.

## Results

**Fig. 1:**
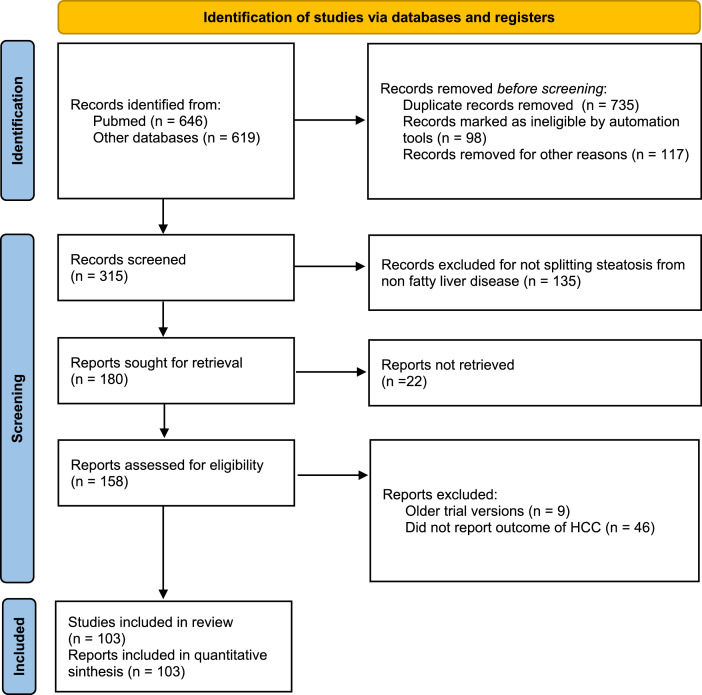
flow diagram of included studies.

A total of 1265 citations were identified. Overall, 103 studies were eligible for inclusion in the present meta-analysis (Figure 1; Table 1; Suppl. File 1). Thus, a total of 948 217 participants with NAFLD were evaluated between 1992 and 2021.

**Table 1:** Characteristics of included studies.

Author/year	Type of study	Country	Median follow up (months)	N° pts with NAFLD (all pts)	Steatosis only %	Steatohepatitis (NASH) %	Cirrosis %	Hepatitis B/C %	Diagnosis (radiological) %	Diagnosis: biopsy %
**Aigelsreiter/2016**	Retrospective	Germany	141	47	36.7	15.6	-	-	-	100
**Alexander/2019**	Retrospective	Europe	39.3	136703	68.3	2	0.4	-	-	-
**Alvarez/2020**	Retrospective	US	324	4355	-	-	-	-	100	-
**Amarapurkar/2008**	Prospective	India	-	585	-	7	17.8	19/14.2	100	-
**Ampuero/2015**	Cross sectional	Spain	-	34	23.5	76.5	70.5	-	-	100
**Arase/2012**	Retrospective	Japan	98.4	1600	-	-	-	0	100	-
**Asahina/2013**	Retrospective	Japan	73.2	431*	-	-	-	100	-	100
**Ascha/2010**	Retrospective	Lebanon	32.4	195	100	100	100	12.8	-	100
**Asfari/2020**	Cross sectional	US	-	218950	100	100	8.2	2.6	-	-
**Bengtsson/2019**	Retrospective	Sweden	16.2	225	-	-	63	0	-	-
**Best/2020**	Prospective	Japan	167	392	-	-	-	-	-	-
**Beste/2015**	Retrospective	US	-	1029	-	-	-	-	100	-
**Bhala/2011**	Prospective	UK	85.6	247	-	-	100	-	-	-
**Carr/2018**	Retrospective	Italy	-	61	-	-	80	0	-	100
**Chan/2017**	Retrospective	China	79.9	107	100	-	-	100	-	100
**Chen CL/2014**	Case control	Taiwan	-	50	-	-	-	100	100	-
**Cho/2011**	Retrospective	Korea	-	54	-	-	-	50	100	-
**Choi/2020**	Retrospective	Canada	120	185	-	100	93	100	-	100
**Chuma/2008**	Retrospective	Japan	122	75	100	-	-	100	-	100
**Cotrim/2011**	Retrospective	Brazil	-	1280	42	58	27	0	-	100
**D'Ambrosio/2018**	Prospective	Italy	120	5	100	-	-	100	-	100
**Dal Bello/2010**	Retrospective	Italy	36	33**	-	-	-	-	-	100
**Doycheva/2019**	Retrospective	US	-	1925	100	100	0	0	-	-
**Dugum/2015**	Retrospective	US	40	838	100	100	0	0	0	100
**Dunn/2013**	Retrospective	US	-	233	100	9	4	0	0	100
**Ekstedt/2015**	Retrospective	Sweden	396	229	-	100	10	0	68	32
**El-derany/2020**	Prospective	Egypt	-	134	100	100	0	0	0	100
**Ertle/2011**	Retrospective	Germany	-	36	100	100	49	0	0	100
**Grimaudo/2020**	Prospective	Italy	64.6	471	100	76.2	34.3	0	0	100
**Hamoir/2021**	Prospective	Belgium	13	16	100	-	100	100	0	100
**Hashimoto/2009**	Prospective	Japan	40.3	382	100	100	100	0	0	100
**Hayashi/2016**	Retrospective	Japan	52.7	544	22.7	-	38.2	-	-	100
**Hernandez-Alejandro/2012**	Retrospective	Canada	-	17	-	100	-	-	100	100
**Hester/2019**	Cross sectional	US	-	2820	-	-	46.9	28.9	-	-
**Hsiang/2014**	Retrospective	New Zeland	47	122	-	-	100	59.6	22.1	21.2
**Huang MY/2017**	Retrospective	Taiwan	72	263	-	-	2.4	2.1	-	-
**Huang Y/2020**	Retrospective	Australia	54	1597	-	-	.	70	-	-
**Hui/2003**	Prospective cohort	Australia	60	23	-	100	-	-	-	100
**Ioannou/2019**	Retrospective	US	44	7068	-	-	100	0	-	-
**Jain/2012**	Retrospective	India	-	47	-	-	100	-	-	100
**Ji/2021**	Prospective	China	48	1241	25.5	-	100	100	-	100
**Kai/2017**	Retrospective	Japan	67	10	-	-	-	0	-	100
**Kanwal/2018**	Retrospective cohort	US	108	296707	-	-	1.4	-	-	-
**Kaplan/2019**	Retrospective	US	30	11306	-	-	-	-	-	-
**Kawamara/2011**	Retrospective	Japan	68	6508	-	-	-	0	100	-
**Kim/2018**	Retrospective	Korea	12	8721	-	-	-	0	100	100
**Kodama/2013**	Prospective	Japan	50	72	-	100	100	0	100	100
**Kumar.2005**	Prospective	Australia	26.2	25	76	-	25	100	100	100
**Kurosaki/2010**	Pospective	Japan	54	1279	100	-	-	100	100	100
**Lee/2016**	Prospective	Korea	45.2	24	100	-	-	100	100	100
**Li/2021**	Prospective	US	140	1079	100		2.5	100	100	100
**Lim/2020**	Retrospective	Singapore	111	185	100	-	10.3	100	100	100
**Lin/2021**	Retrospective	Taiwan	65	369	100	-	-	100	-	100
**Malik/2009**	Retrospective	US	60	98		77.6	22.4	0	-	72.4
**Marot/2017**	Retrospective	Switzerland/Belgium	-	78	-	-	-	-	-	-
**Mittal/2015**	Retrospective	USA	-	120	100	0	58.3	-	-	53.4
**Nakajima/2011**	Retrospective	Japan	-	92	34.8	59.8	5.4	0	100	100
**Nirei/2017**	Retrospective	Japan	-	170	100	-	100	100	0	100
**Nkontchou/2011**	Retrospective	France	66	340	100	-	100	100	-	100
**Ogawa/2020**	Retrospective	Japan	60	290	0	100	76	100	-	100
**Ohata/2003**	Retrospective	Japan	76.5	90	76	100	100	100	-	100
**Paradis/2009**	Retrospective	France	-	60	-	-	-	-	-	100
**Pekow/2006**	Retrospective	US	-	23	100	0	100	100	-	100
**Peleg/2019**	Retrospective	Israel	72	241	100	0	19.3	100	-	100
**Petit/2013**	Retrospective	France	NA	141	-	-	100	-	-	-
**Phan/2019**	Retrospective	US	-	28	-	-	89	-	-	100
**Pinyopornpanish/2021**	Retrospective	US	13.8	346	-	-	14	-	-	-
**Reddy/2012**	Retrospective	US	50	52	-	100	-	-	-	100
**Sadler/2017**	Retrospective	US/Canada	56.1	60	0	100	0	0	100	-
**Safcak/2021**	Retrospective	Slovakia	-	54	-	-	85.2	0	100	-
**Sanyal/2010**	Retrospective	US	-	3933	-	-	-	4.5/31.1	-	-
**Schutte/2014**	Retrospective	Germany	-	43	-	100	-	-	-	-
**Sharma/2018**	Retrospective	UK/Canada	-	111	-	-	100	-	-	-
**Shibahara/2014**	Retrospective	Japan	-	106	-	38.7	36.8	11.3/45.3	-	100
**Shimomura/2017**	Prospective observational	Japan	-	69	14	55	-	0	-	100
**Shingina/2019**	Retrospective	US	-	182368	-	9	-	38	-	-
**Simon/2021**	Retrospective	Sweden	-	10568	67.2	27.2	5.6	0	-	100
**Su/2015**	Retrospective	China	69.8	74	-	-	-	93	-	100
**Takahashi/2011**	Prospective cohort	Japan	-	13	100	-	-	100	-	100
**Takuma/2007**	Retrospective	Japan	45.1	25	100	-	-	65.9	-	-
**Tanaka/2013**	Retrospective	Japan	-	49	26.5	73.5	-	0	-	100
**Tateishi/2015**	Retrospective	Japan	31	596	-	-	61.7	26.7	-	-
**Thuluvath/2018**	Retrospective	US	-	11302	-	100	-	100	-	-
**Tokushige/2010**	Prospective observational	Japan	35.4	34	-	100	-	0	-	61.7
**Tokushige/2013**	Retrospective	Japan	-	292	-	-	72	83	-	100
**Van meer/2015**	Retrospective	The Netherlands	11	176	-	-	97	37	-	100
**Van Meer/2016**	Retrospective	The Netherlands	12	181	-	-	81	38	-	100
**Viganò/2015**	Retrospective	Italy	44.6	96	45.8	25	22.9	*0*	-	100
**Wakai/2011**	Retrospective	Japan	87	17	-	47	75	92	-	100
**Walker/2016**	Retrospective	US	-	204	-	-	100	74	-	100
**Wang /2021**	Retrospective	China	-	17528	-	-	-	-	100	-
**Wild/2018**	Retrospective	UK	56.4	1452	-	-	-	-	-	19
**Wong/2019**	Retrospective	US	-	138	0	100	0	64	-	100
**Yatsuji/2008**	Prospective	Japan	-	68	-	100	-	-	-	100
**Wu/2011**	Retrospective	China	53.1	355	100	-	-	91.9	100	100
**Wu/2018**	Retrospective	US/Asia	-	113	-	100	-	-	100	100
**Yang 2016**	Retrospective	US	38	173	-	-	100	44	100	100
**Yen/2017**	Retrospective	China	97.3	140	100	-	100	100	100	100
**Yoon/2020**	Prospective	Korea	74.9	88	-	100	39.8	100	100	100
**Younossi/2019**	Retrospective	US	-	2690	-	100	-	-	100	100
**Yu/2008**	Prospective	Taiwan (China)	176.4	1850	-	-	22.1	100	100	100
**Zhang/2016**	Prospective	China	-	7	-	-	75.3	100	100	100
**Zheng/2017**	Retrospective	US	23	141	100	-	25	45	100	100

NAFLD, non-alcoholic fatty liver disease; NASH, non alcoholic steato-hepatitis; *. severe steatosis only; °. grade 2-3 only; **. grade 3 only; °°. higher fibrosis only

### Characteristics of the Included Studies

All of the studies were observational and not intervention studies, 77 were retrospective series, 22 were prospective studies, 1 was a case control-study, and 3 were cross-sectional studies. Among the included studies, 42 were conducted in Asia, with the remaining having been conducted in Europe, Australia, or the United States. The median follow-up ranged from 11 to 396 months (mean 85). The mean NOS score was 6.4. A total of 209 110 cases of HCC were described, for a pooled incidence of 22%.

### NAFLD and HCC Risk

A total of 43 papers evaluated the risk of HCC over time in patients with steatosis/NASH with or without cirrhosis. The risk of HCC was 1.88 (95% CI, 1.46, 2.42), *P* < .01 (Figure 1). After excluding 5 studies in which HRs were calculated according to univariate analysis, the risk was even higher (HR = 2.19 [95% CI, 1.67, 2.87]; *P* < .01). This means that NAFLD is an independent risk factor for HCC development. The risk was unchanged after meta-regression analysis was performed according to the rate of steatosis, NASH, cirrhosis, viral hepatitis, and the follow-up duration. Regarding ethnicity, results were not significant for the studies conducted in Asian countries (HR = 1.43 [95% CI, 0.94-2.17]; *P* = .1) but were for studies conducted in Western countries (HR = 2.63 [95% CI, 1.79-3.87]; *P* < .01). In papers with poor- to moderate-quality NOS scores, the risk of HCC was not significant, but the risk was significant in good-quality papers (with longer/known follow-up periods; HR = 3.18 [95% CI, 1.98-5.11]; *P* < .01).

### HCC Prognosis According to Steatosis

**Fig. 2:**
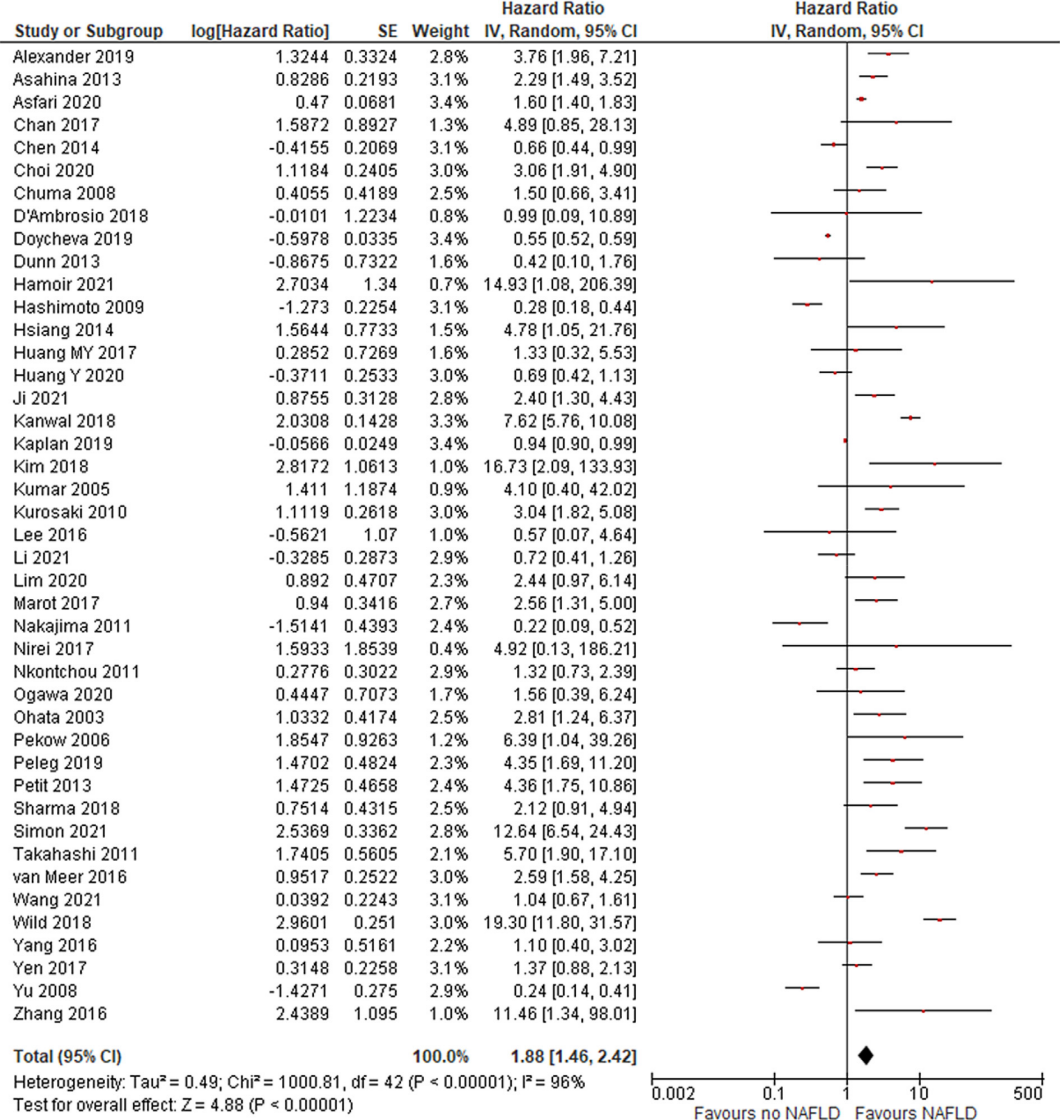
risk of HCC in patients with NAFLD.

**Fig. 3:**
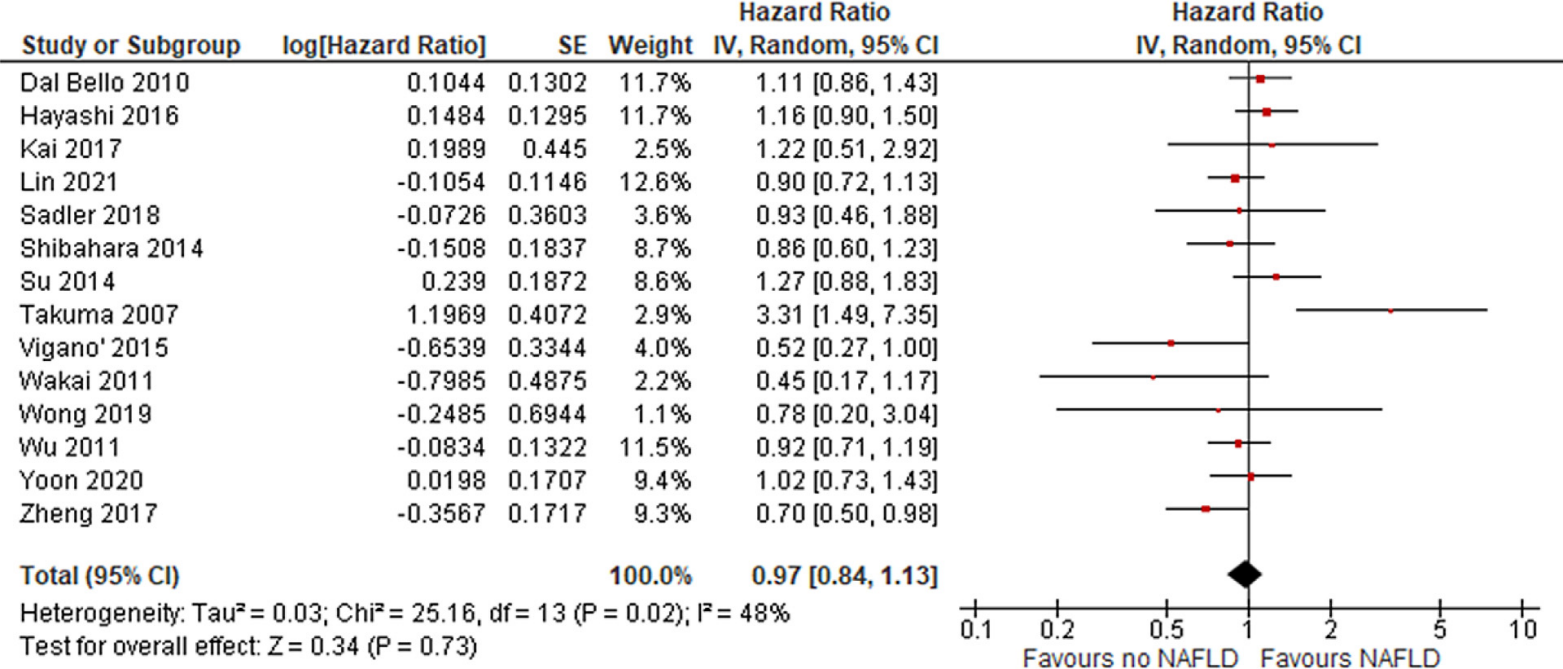
DFS in patients with HCC and NAFLD.

In total, 14, 8, and 24 studies evaluated HCC prognosis according to NAFLD state in terms of DFS, CSM, and OS, respectively. Overall, no difference was found for recurrence (DFS HR = 0.97 [95% CI, 0.84, 1.13]; = *P* = .73), CSM (HR = 2.16 [95% CI, 0.85, 5.5]; P = .1; Figure 2), or OS (HR = 1.02 [95% CI, 0.86, 1.21]; *P* = .84; Figure 3), revealing that outcome of HCC is not more unfavorable in patients with NAFLD.

### Publication Bias

Evidence of publication bias regarding risk of HCC meta-analysis was observed based on the results of a funnel plot (*P* < .01) but not with Egger's test (*P* = .18).

## Discussion

Through a meta-analysis of published literature, we evaluated the risk of HCC in patients with NAFLD in the general population. We confirmed that NAFLD was independently associated with an 88% increased risk of HCC, as compared to no NAFLD, in a series of 103 studies published across 3 decades. In a similar meta-analysis published in 2018, Stine et al. found that the risk of HCC was significantly increased only in patients with noncirrhotic NASH but not in the whole NASH population (with or without cirrhosis) [6]. However, a meta-regression analysis adjusted for the rate of steatosis/NASH and fibrosis did not confirm these findings. Steatohepatitis was also associated with an increased risk of intrahepatic cholangiocarcinoma and colorectal cancer [7],[8]. Similar causative factors such as diabetes, overweight, or hepatitis C may be responsible for this association. In fact, approximately 30% to 40% of incident HCC cases are associated with metabolic syndrome. Type 2 diabetes is also a risk factor for NAFLD and increases HCC incidence [9].

NAFLD mouse models showed altered compositions of their gut microbiome. NAFLD HCC patients had increased levels of IL-13, which can activate myeloid-derived suppressor cells and promote tumor progression by inhibiting cancer immunity [10].

Another mechanism of NAFLD-associated HCC is PNPLA3 polymorphisms, which are associated with general NAFLD progression, by enhancing inflammatory signals, including in the IL-6/STAT3 and CCL5 pathways [11].

Nonalcoholic fatty liver disease (and NASH-related cirrhosis in particular) is an emerging risk factor for HCC in Western countries. However, risk of HCC was increased in Western populations but not Asian populations in subgroup analysis. The indication for liver transplant is increased more than 11-fold worldwide [12]. It is rare, however, to observe HCC in the absence of liver inflammation or cirrhosis [13]. In the present meta-analysis, in fact, the papers included almost all subjects with NASH/cirrhosis, with or without viral hepatitis. We found that steatosis/NASH was an independent risk factor for HCC, as compared to no steatosis/no NASH (more than doubling the risk). Conversely, NAFLD-associated HCC was not linked with a poorer prognosis, as compared to non-NAFLD-related cancers. It appears that steatosis or NASH may exert a somewhat protective effect on the HCC course. Even in the general population, NASH patients without or with minimal fibrosis, but not those with higher levels of fibrosis, have a better prognosis in terms of overall mortality [14], [15]. Even in the NHANES cohort, patients with NASH but not advanced fibrosis had a lower risk of death [16]. However, in our review, HCC-related mortality but not overall mortality was (not significantly) higher in patients with NAFLD. This may be due to the high rates of fibrosis and viral hepatitis C in our cohorts.

These observations highlight that patients with NASH with or without initial fibrosis may need intensive surveillance and treatment to slow or revert fibrosis evolution and cancer transformation. In patients with NAFLD and cirrhosis, in fact, the management is similar to that for cirrhosis due to other causes and includes screening for hepatocellular carcinoma, lifestyle interventions, and evaluation for liver transplantation, for patients with decompensated cirrhosis or HCC.

Treatment of NAFLD-associated HCC is not different from that of HCC related to other causes. Instead, the role of immunotherapy, which provides a better outcome for advanced HCC than antiangiogenetic drugs do, has been questioned in NASH patients. In preclinical models of NASH-induced HCC, the delivery of immunotherapy-targeting programmed death-1 (PD1), in fact, expanded activated CD8+PD1+ T-cells within tumors but did not lead to tumor regression, indicating that tumor immune surveillance was impaired. These observations seem to confirm that NASH-associated HCC might be less responsive to immunotherapy, probably due to NASH-related aberrant T-cell activation causing tissue damage that leads to impaired immune surveillance [17].

Our meta-analysis suffers several limitations, including regarding its inclusion criteria (patients with both steatosis or NASH and various degrees of fibrosis), confounders due to viral hepatitis coinfection, duration of follow-up, race, and NAFLD diagnosis. However, this is the largest meta-analysis to have been performed on studies on how NAFLD affects the risk and prognosis of HCC.

We can conclude that NAFLD, with or without fibrosis, is a major risk factor for the development of HCC. Despite this, overall mortality and CSM are not significantly increased when HCC is diagnosed. Despite the heterogeneity of the included literature and the degree of NAFLD of our populations, these subjects deserve similar follow-up and management like patients with other chronic liver diseases receive.

**Fig. 4:**
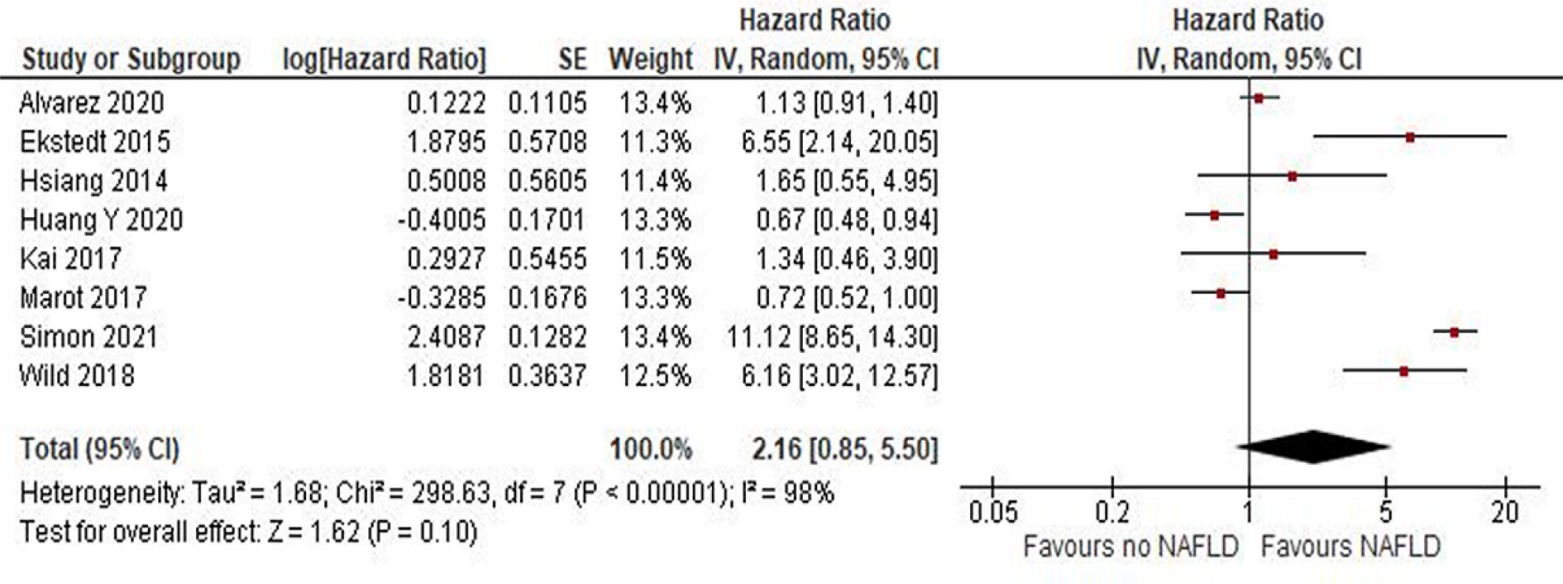
CSM in patients with HCC and NAFLD.

**Fig. 5:**
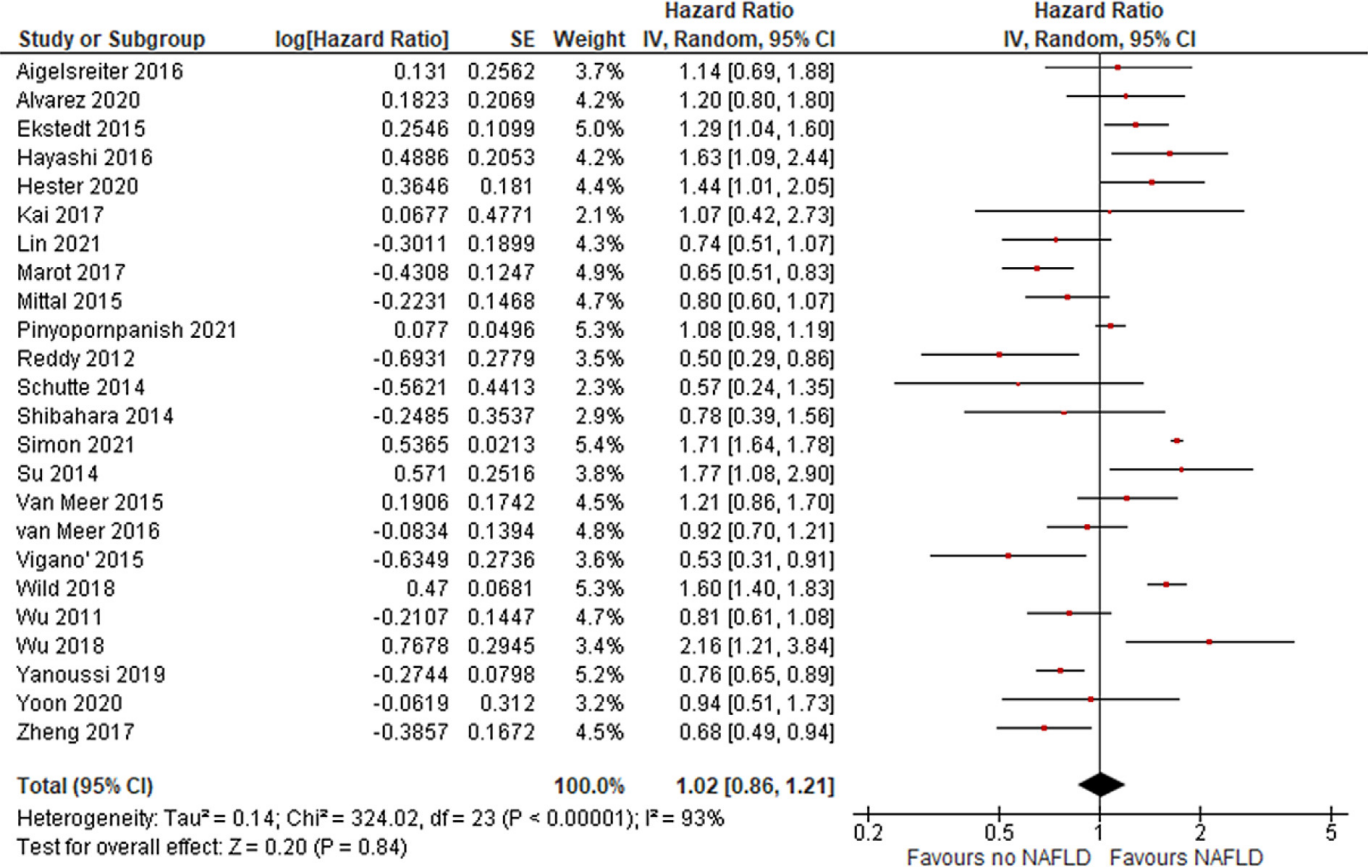
OS in patients with HCC and NAFLD.

Figs. 4, 5 and Table 2

**Table 2:** Frequence and outcome of HCC in patients with NAFLD.

Author/year	HCC n/%	HCC risk: HR or OR (95%CI)	Type of analysis	HCC DFS: HR (95%CI)	Type of analysis	Cancer mortality HR (95%CI)	Type of analysis	HCC OS: HR (95%CI)	Type of an alysis	NOS score
**Aigelsreiter/2016**	-	-	-	1.07 (0.67-1.69)	UVA	-	-	1.14 (0.69-1.89)	UVA	8
**Alexander/2019**	176/0.1	3.76 (1.96-7.20)°	MVA	-	-	-	-	-	-	6
**Alvarez/2020**	-	-	-	-	-	1.13 (0.91-1.39)	MVA	1.2 (0.8-1.34)	MVA	9
**Amarapurkar/2008**	54/9.2	-	-	-	-	-	-	-	-	5
**Ampuera/2015**	7/20.6	-	-	-	-	-	-	-	-	5
**Arase/2012**	10/6	-	-	-	-	-	-	-	-	8
**Asahina/2013**	-	2.29 (1.49-3.50)^	MVA	-	-	-	-	-	-	8
**Ascha/2010**	25/12.8	-	-	-	-	-	-	-	-	7
**Asfari/2020**	10947/0.5	1.6 (1.4-1.9)°	MVA	-	-	-	-	-	-	6
**Bengtsson/2019**	225/14.4	-	-	-	-	-	-	-	-	6
**Best/2020**	29/7.1	-	-	-	-	-	-	-	-	8
**Beste/2015**	1029/-	-	-	-	-	-	-	-	-	9
**Bhala/2011**	6/2.4	-	-	-	-	-	-	-	-	8
**Carr/2081**	16/-	-	-	-	-	-	-	-	-	5
**Chan/2017**	11/4.1	6.58 (0.9-46.8)* 3.2 (0.8-12.3)°	UVA	-	-	-	-	-	-	9
**Chen/2014**	50/-	0.66 (0.44–0.99)	MVA	-	-	-	-	-	-	5
**Cho/2011**	54/-	-	-	-	-	-	-	-	-	5
**Choi/2020**	16/8.6	3.06 (1.91-4.91)°	UVA	-	-	-	-	-	-	9
**Chuma/2008**	35/33.7	1.5 (0.66–3.4)^	MVA	-	-	-	-	-	-	9
**Cotrim/2011**	3/0.2	-	-	-	-	-	-	-	-	5
**D'Ambrosio/2018**	5/-	0.99 (0.09-10.89)	UVA	-	-	-	-	-	-	9
**Dal Bello/2010**	207/-	-	-	1.11 (0.86-1.42)	UVA	-	-	-	-	6
**Doycheva/2019**	1925/-	0.55 (0.52-0.59)	MVA	-	-	-	-	-	-	5
**Dugum/2015**	838/-	-	-	-	-	-	-	-	-	7
**Dunn/2013**	2/1	0.42 [0.10, 1.76]	UVA	-	-	-	-	-	-	5
**Ekstedt/2015**	-	-	-	-	-	6.55 (2.14-20)	UVA	1.24 (1.04-1.59)	UVA	9
**El-derany/2020**	55/-	-	-	-	-	-	-	-	-	5
**Ertle/2011**	36/-	-	-	-	-	-	-	-	-	5
**Grimaudo/2020**	13/2.7	-	-	-	-	-	-	-	-	8
**Hamoir/2021**	3/18.7	14.93 (1.08-206.39)	MVA	-	-	-	-	-	-	6
**Hashimoto/2009**	34/8.9	0.28 (0.18-0.45)	UVA	-	-	-	-	-	-	6
**Hayashi/2016**	544/-	-	-	1.17 (0.90-1.53)	MVA	-	-	1.63 (1.09-2.52)	MVA	7
**Hernandez-Alejandro/2012**	17/-	-	-	-	-	-	-	-	-	5
**Hester/2019**	2820/-	-	-	-	-	-	-	1.44 (1.01-2.07)	MVA	8
**Hsiang/2014**	-	4.78 (1.05-21.79)	MVA	-	-	1.11 (1.01-1-13)	MVA	-	-	6
**Huang/2017**	-	1.33 (0.32-5.53)	MVA	-	-	-	-	-	-	5
**Huang/2020**	226/2	1.69 (1.43-2)	MVA	-	-	0.67 (0.48-0.95)	MVA	-	-	7
**Hui/2003**	0/0	-	-	-	-	-	-	-	-	7
**Ioannou/2019**	690/54	-	-	-	-	-	-	-	-	5
**Jain/2012**	8/17	-	-	-	-	-	-	-	-	
**Ji/2021**	54/4.3	2.4 (1.3-4-2)	MVA	-	-	-	-	-	-	6
**Kai/2017**	83/100	-	-	1.22 (0.51-2.89)	UVA	-	-	1.07 (0.42-2.73)	UVA	6
**Kanwal/2018**	367/0.12	7.62 (5.76-10.09)	MVA	-	-	-	-	-	-	9
**Kaplan/2019**	-	0.94 (0.90-0.99)	MVA	-	-	-	-	-	-	6
**Kawamura/2011**	16/0.25	-	-	-	-	-	-	-	-	5
**Kim/2018**	13/8721	16.73 (2.09-133.85)	MVA	-	-	-	-	-	-	6
**Kodama/2013**	16/-	-	-	-	-	-	-	-	-	7
**Kumar/2005**	25/-	4.1 (0.4-39)*	MVA	-	-	-	-	-	-	8
**Kurosaki/2010**	68/-	3.04 (1.82-5.06)	MVA	-	-	-	-	-	-	7
**Lee/2016**	-	0.57 (0.07-4.74)	UVA	-	-	-	-	-	-	7
**Li/2021**	40/3.74	0.72 (0.41-1.30)	MVA	-	-	-	-	-	-	9
**Lim/2020**	27/289	2.44 (0.97-6.1)	MVA	-	-	-	-	-	-	9
**Lin/2021**	369/-	-	-	0.9 (0.72-0.13)	UVA	0.74 (0.51-1.07)	UVA	-	-	8
**Malik/2009**	17/17.3	-	-	-	-	-	-	-	-	7
**Marot/2017**	12/15	2.56 (1.31-5.00)	MVA	-	-	-	-	-	-	5
**Mittal/2015**	120/-	-	-	-	-	-	-	0.8 (0.6-1.0)	MVA	5
**Nakajima/2011**	14/15.2	0.22 (0.09-0.61)	UVA	-	-	-	-	-	-	5
**Nirei/2017**	12/7	4.92 (0.13-186)*	MVA	-	-	-	-	-	-	5
**Nkontchou/2011**	96/28	1.32 (0.73-2.39)*	-	-	-	-	-	-	-	7
**Ogawa/2020**	16/0.5	1.56 (0.39-6.24)*	UVA	-	-	-	-	-	-	7
**Ohata/2003**	-	2.81 (1.24-6.37)^	MVA	-	-	-	-	-	-	8
**Paradis/2009**	60/-	-	-	-	-	-	-	-	-	5
**Pekow/2006**	32/-	6.39 (1.04-39.3)*	MVA	-	-	-	-	-	-	5
**Peleg/2019**	14/5.8	4.35 (1.69-11.2)	MVA	-	-	-	-	-	-	8
**Phan/2019**	3/-	-	-	-	-	-	-	-	-	5
**Pinyopornpanish/2021**	346/-	-	-	-	-	-	-	1.08 (0.98-1.28)	MVA	6
**Reddy/2012**	52/-	-	-	-	-	-	-	0.50 (0.29-0.88)	MVA	6
**Sadler/2017**	60/-	-	-	0.93 (0.45-1.92)	UVA	-	-	-	-	8
**Safcak/2021**	54/-	-	-	-	-	-	-	-	-	5
**Sanyal/2010**	2578/58.5	-	-	-	-	-	-	-	-	5
**Schutte/2014**	43/-	-	-	-	-	-	-	0.57 (0.24-1.34)	UVA	5
**Sharma/2018**	8/3.5	2.12 (0.91-4.92)	MVA	-	-	-	-	-	-	5
**Shibahara/2014**	106/-	-	-	0.87 (0.62-1.23)^0.83 (0.55-1.25)°	UVA	-	-	0.80 (0.41-1.56)^0.75 (0.34-1.64)°	UVA	5
**Shimomura/2017**	-	-	-	-	-	-	-	-	-	6
**Shingina/2019**	2181/13	-	-	-	-	-	-	-	-	6
**Simon/2021**	186/-	-	-	-	-	-	-	-	-	6
**Su/2015**	74/-	-	MVA	-	-	-	-	-	-	7
**Takahashi/2011**	6/46.2	5.7 (1.9-17.1)	MVA	-	-	-	-	-	-	7
**Takuma/2007**	25/-	-	-	3.31 (1.49-7.41)	MVA	-	-	-	-	7
**Tanaka/2013**	6/16.7	-	-	-	-	-	-	-	-	5
**Tateishi/2015**	596/-	-	-	-	-	-	-	**-**	-	6
**Thuluvath/2018**	2166/19	-	-	-	-	-	-	-	-	5
**Tokushige/2010**	34/-	-	-	-	-	-	-	-	-	7
**Tokushige/2013**	292/-	-	-	-	-	-	-	-	-	5
**Van Meer/2015**	176/-	-	-	-	-	-	-	-	-	6
**Van Meer/2016**	181/-	2.59 (1.58-4.26)	MVA	-	-	-	-	-	-	6
**Viganò/2015**	96/-	-	-	0.55 (0.36-0.85)	MVA	-	-	0.53 (031-0.91)	MVA	7
**Wakai/2011**	17/-	0.45 (0.17-1.17)	MVA	-	-	-	-	-	-	8
**Walker/2016**	204/-	-	-	-	-	-	-	-	-	5
**Wang/2021**	39/0.2	1.07 (0.73-1.58)	UVA	-	-	-	-	-	-	5
**Wild/2018**	19/-	19.3 (11.8-31.4)	-	-	-	6.16 (3.02-12.6)	MVA	-	-	7
**Wong/2019**	138/-	0.78 (0.20-3.03)	UVA	-	-	-	-	-	-	5
**Yatsuji/2008**	7/10	-	-	-	-	-	-	-	-	5
**Wu/2011**	355/-	-	-	0.92 (0.71-1.19)	MVA	-	-	0.81 (0.61-1.08)	MVA	6
**Wu/2018**	113/-	-	-	-	-	-	-	2.16 (1.21-3.84)	-	5
**Yang/2016**	-	1.10 (0.40-3.02)	MVA	-	-	-	-	-	-	6
**Yen/2017**	140/14.36	1.37 (0.88-2.13)	MVA	-	-	-	-	-	-	8
**Yoon/2020**	196/50	-	-	1.02 (0.73-1.43)	MVA	-	-	0.94 (0.51-1.73)	MVA	8
**Younossi/2019**	2690/-	-	-	-	-	4.17 (3.81-4.56)	MVA	0.76 (0.65-0.89)	MVA	5
**Yu/2008**	-	0.24 (0.14-0.41)	MVA	-	-	-	-	-	-	9
**Zhang/2016**	6/1.38	11.46 (1.34-98.01)	MVA	-	-	-	-	-	-	5
**Zheng/2017**	141/-	-	-	0.70 (0.50-0.98)	MVA	-	-	0.68 (0.49-0.94)	MVA	6

HCC, hepatocellular carcinoma; HR, hazard ratio; OR, odds ratio; CI, confidence interval; DFS, disease-free survival; OS, overall survival; UVA, univariate analysis; MVA, multivariate analysis; *, grade 2-3 vs 0; °, steatohepatitis; ^, steatosis grade 1-3 vs 0, **, composite outcome of cancer incidence and mortality.

## Declaration of Competing Interest

None to declare
